# Epigenetic reprogramming and aberrant expression of PRAME are associated with increased metastatic risk in Class 1 and Class 2 uveal melanomas

**DOI:** 10.18632/oncotarget.10962

**Published:** 2016-07-30

**Authors:** Matthew G. Field, Michael A. Durante, Christina L. Decatur, Bercin Tarlan, Kristen M. Oelschlager, John F. Stone, Jeffim Kuznetsov, Anne M. Bowcock, Stefan Kurtenbach, J. William Harbour

**Affiliations:** ^1^ Bascom Palmer Eye Institute, Sylvester Comprehensive Cancer Center and Interdisciplinary Stem Cell Institute, University of Miami Miller School of Medicine, Miami, FL, USA; ^2^ Castle Biosciences, Inc., Friendswood, TX, USA; ^3^ National Heart and Lung Institute, Imperial College London, London, UK

**Keywords:** PRAME, preferentially expressed antigen in melanoma, uveal melanoma, DNA methylation, chromosomal instability

## Abstract

**Background:**

We previously identified *PRAME* as a biomarker for metastatic risk in Class 1 uveal melanomas. In this study, we sought to define a threshold value for positive *PRAME* expression (PRAME+) in a large dataset, identify factors associated with *PRAME* expression, evaluate the prognostic value of *PRAME* in Class 2 uveal melanomas, and determine whether *PRAME* expression is associated with aberrant hypomethylation of the *PRAME* promoter.

**Results:**

Among 678 samples analyzed by qPCR, 498 (73.5%) were PRAME- and 180 (26.5%) were PRAME+. Class 1 tumors were more likely to be PRAME-, whereas Class 2 tumors were more likely to be PRAME+ (*P* < 0.0001). *PRAME* expression was associated with shorter time to metastasis and melanoma specific mortality in Class 2 tumors (*P* = 0.01 and *P* = 0.02, respectively). In Class 1 tumors, *PRAME* expression was directly associated with *SF3B1* mutations (*P* < 0.0001) and inversely associated with *EIF1AX* mutations (*P* = 0.004). *PRAME* expression was strongly associated with hypomethylation at 12 CpG sites near the *PRAME* promoter.

**MATERIALS AND METHODS:**

Analyses included *PRAME* mRNA expression, Class 1 versus Class 2 status, chromosomal copy number, mutation status of *BAP1*, *EIF1AX*, *GNA11*, *GNAQ* and *SF3B1*, and genomic DNA methylation status. Analyses were performed on 555 de-identified samples from Castle Biosciences, 123 samples from our center, and 80 samples from the TCGA.

**Conclusions:**

*PRAME* is aberrantly hypomethylated and activated in Class 1 and Class 2 uveal melanomas and is associated with increased metastatic risk in both classes. Since PRAME has been successfully targeted for immunotherapy, it may prove to be a companion prognostic biomarker.

## INTRODUCTION

Uveal melanoma is the most common primary cancer of the eye and the second most common form of melanoma. Due to a high rate of metastasis, much research has focused on the development of biomarkers to predict metastatic risk. Previously, we described a gene expression profile that could be performed on a fine needle biopsy of the primary tumor that accurately predicted metastasis [1]. Tumors with the Class 1 profile have a low metastatic risk, whereas those with the Class 2 profile have a high metastatic risk. Consequently, a 15 gene array (12 discriminating genes and 3 control genes) was developed and prospectively validated [2, 3]. This assay is now available commercially as the DecisionDx-UM^™^ test (Castle Biosciences), which has been independently validated [4] and is widely used to stratify patients for metastatic surveillance and to identify high risk patients for adjuvant therapy trials [5].

While the vast majority of metastatic events in uveal melanoma arise from Class 2 tumors, a small subset of Class 1 tumors also give rise to metastasis. We found that the expression of two of the 12 discriminating genes on the array (*CDH1* and *RAB31*) could be used to identify Class 1 tumors that may have increased metastatic risk. Class 1 tumors with low expression of these genes and very low predicted metastatic risk were called Class “1A,” whereas those with high expression and higher predicted metastatic risk were called Class “1B.” In our efforts to further improve the prognostic accuracy of the gene array platform, we conducted a genome wide search for new biomarkers and found that mRNA expression of the cancer-testis antigen Preferentially Expressed Antigen in Melanoma (*PRAME*) was an accurate biomarker for metastasis in Class 1 tumors [6]. In that initial study, we found that any detectable mRNA expression of *PRAME* above baseline was associated with increased metastatic risk. Limitations of that study included a relatively small number of tumor samples that were biased towards larger tumor size.

To date, there have been five common driver mutations identified in uveal melanoma: *BAP1*, *EIF1AX*, *GNA11*, *GNAQ* and *SF3B1* [7–11]. Mutations in *BAP1*, *SF3B1* and *EIF1AX* are almost mutually exclusive and are associated with high, intermediate and low metastatic risk, respectively [6, 12]. Also, *SF3B1* mutations were found to be associated with *PRAME* expression [6].

The purpose of the present study was to study *PRAME* expression in a much larger number of Class 1 and, for the first time, in Class 2 uveal melanomas spanning the true range of tumor sizes encountered in clinical practice. We sought to define a threshold value for calling a tumor sample positive for *PRAME* expression (PRAME+), compare *PRAME* expression to the 1A/1B designation in Class 1 tumors, identify clinical and molecular factors associated with *PRAME* expression, evaluate the prognostic value of *PRAME* expression in Class 2 tumors, and determine whether *PRAME* expression in uveal melanoma is correlated with promoter hypomethylation.

## RESULTS

**Figure 1:**
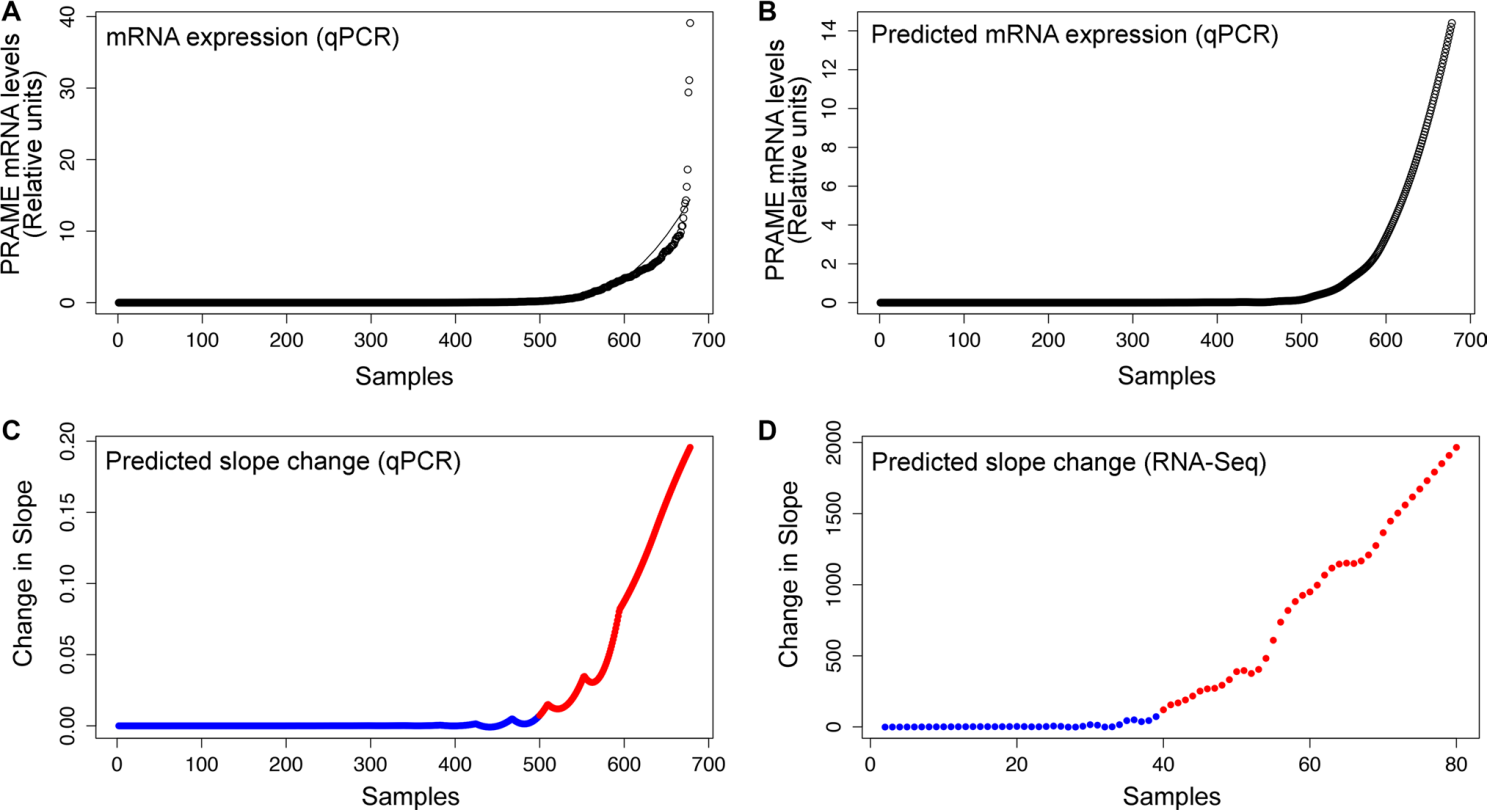
Defining the threshold for PRAME+ expression status (**A**) *PRAME* mRNA expression plotted from lowest to highest expression for 678 uveal melanoma samples measured by qPCR with a LOESS model (second degree, family = ”Gaussian”, spanning 0.4, fitting by least-squares). (**B**) Predicted *PRAME* mRNA expression for an additional “hypothetical” 678 samples based on the LOESS model. (**C**) Predicted slope change between each of these predicted points. (**D**) The same process depicted in panels A–C was repeated separately for the RNA-Seq data from 80 TCGA uveal melanoma samples in order to generate a predicted slope change plot. For both datasets, the threshold for PRAME+ (red) was defined as the point where the slope sustainably rose above baseline (blue).

To evaluate the spectrum of *PRAME* mRNA expression and to establish a threshold for positive *PRAME* expression in primary uveal melanoma, we analyzed qPCR data from 678 tumor samples, including 123 of our samples and 555 de-identified samples submitted from a large number of ocular oncology centers to Castle Biosciences. These samples included 454 (67.0%) Class 1 tumors and 224 (33.0%) Class 2 tumors. Class 1 tumors included 317 (69.8%) Class 1A tumors, 131 (28.9%) Class 1B tumors, and 6 (1.3%) tumors for which 1A/1B information was not available. Whereas most samples showed negligible *PRAME* expression, a subset of samples showed a broad range of *PRAME* expression (Figure 1A). We previously showed that any *PRAME* expression above baseline was associated with increased metastasis in Class 1 tumors and consequently defined any expression above baseline as positive *PRAME* expression (PRAME+) [6]. In this study, we used the same methodology to establish a broadly applicable PRAME+ threshold from qPCR data using a much larger dataset that included both Class 1 and Class 2 tumors, with a majority derived from fine needle biopsy of small and medium sized tumors and a smaller number from large, enucleated specimens that is representative of the actual distribution of tumor sizes encountered in clinical practice (Figure 1B–1C). A similar method was used to determine a PRAME+ threshold using RNA-Seq data from The Cancer Genome Atlas (TCGA) dataset (Figure 1D).

**Figure 2:**
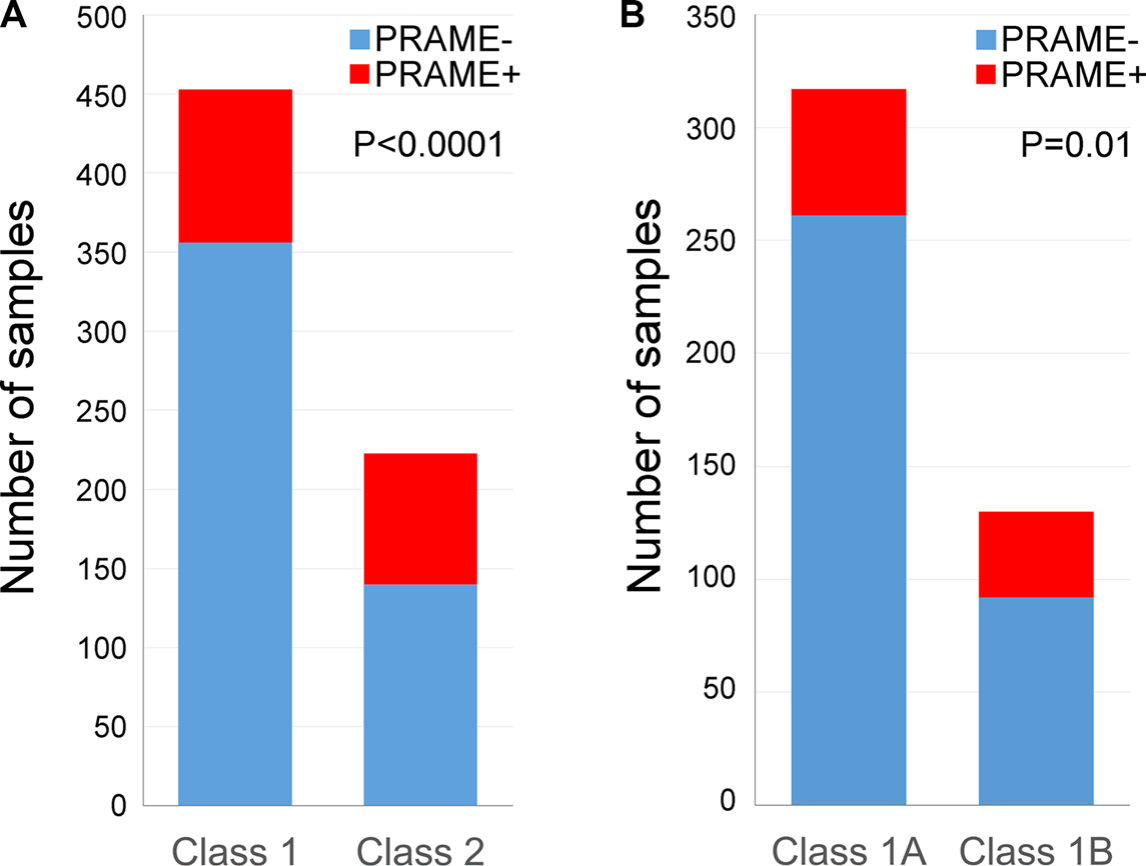
Summary of *PRAME* expression status measured by qPCR (**A**) *PRAME* expression status with respect to gene expression profile classification in 678 uveal melanomas. (**B**) *PRAME* expression status in 454 Class 1 uveal melanomas with respect to 1A/1B sub-classification.

Overall, 498 (73.5%) tumors were PRAME− and 180 (26.5%) were PRAME+. Class 1 tumors were more likely to be PRAME−, whereas Class 2 tumors were more likely to be PRAME+ (Fisher exact test, *P* < 0.0001) (Figure 2A). Among Class 1 tumors, 357 (78.6%) were PRAME− and 97 (21.4%) were PRAME+. Among Class 1A tumors, 261 (82.3%) were PRAME− and 56 (17.7%) were PRAME+. Among Class 1B tumors, 93 (71.0%) were PRAME− and 38 (29.0%) were PRAME+. Class 1A tumors were more likely to be PRAME−, whereas Class 1B tumors were more likely to be PRAME+ (Fisher exact test, *P* = 0.01) (Figure 2B). Among Class 2 tumors, 141 (62.9%) were PRAME− and 83 (37.1%) were PRAME+. Additionally, we determined *PRAME* mRNA status in commonly used UM cell lines: Mel202 and MP41 are PRAME+, whereas 92.1, Mel270, Mel290, and MP46 are PRAME−.

### Association between PRAME and clinical features

Clinical annotations and *PRAME* expression were available for 123 of our uveal melanoma samples (Supplementary Table S1). The only features that were significantly associated with PRAME+ status were larger tumor diameter and thickness (Mann-Whitney test, *P* = 0.01 and *P* = 0.02, respectively). To expand this analysis, we examined the TCGA Research Network dataset which consists of an independent cohort of 80 primary uveal melanoma samples (http://cancergenome.nih.gov/) (Supplementary Table S2). Since *PRAME* expression data were obtained from RNA-Seq analysis in the TCGA dataset, we established a threshold for PRAME+ expression using the same procedure as for qPCR data (Figure 1D). Consistent with our original dataset, PRAME+ status in the TCGA dataset was associated with larger tumor diameter (*P* = 0.02). In both datasets, well-known risk factors for metastasis such as increased patient age, ciliary body involvement, and extrascleral tumor extension were not associated with *PRAME* expression status.

**Figure 3:**
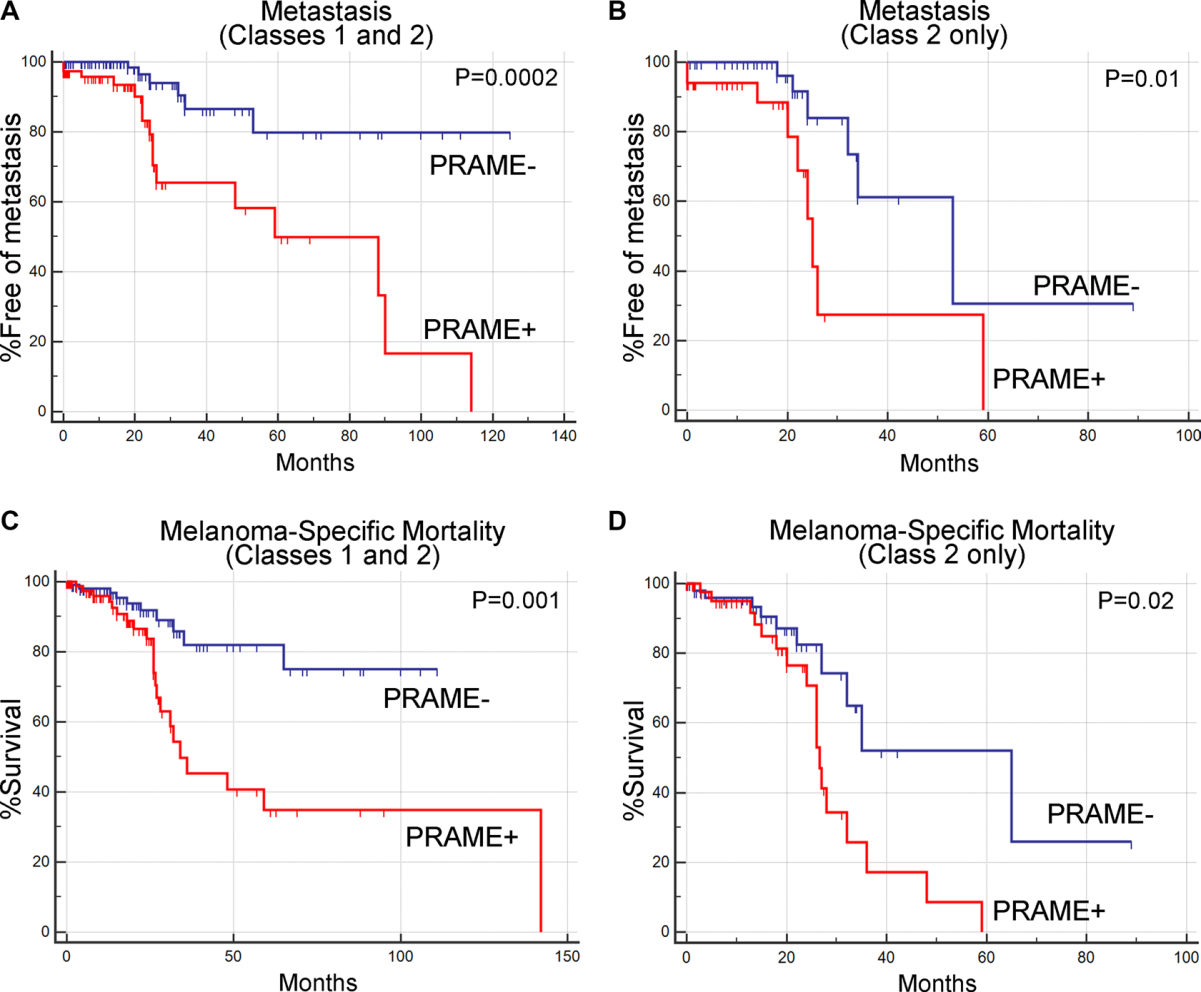
Prognostic significance of *PRAME* expression status in uveal melanoma (**A**) Kaplan-Meier survival plot showing metastasis-free survival for Class 1 and Class 2 tumors combined, with respect to *PRAME* expression status. (**B**) Kaplan-Meier survival plot showing metastasis-free survival for Class 2 tumors only, with respect to *PRAME* expression status. (**C**) Kaplan-Meier plot showing melanoma-specific mortality for Class 1 and Class 2 tumors combined, with respect to *PRAME* expression status. (**D**) Kaplan-Meier survival plot showing melanoma-specific mortality for Class 2 tumors only, with respect to *PRAME* expression status.

We previously showed that *PRAME* expression was associated with increased metastatic risk in Class 1 uveal melanomas [6]. Here, we extend that analysis to determine whether *PRAME* expression is also a biomarker for metastasis in Class 2 tumors. For this analysis, we combined our 123 cases with the 80 cases from the TCGA. Using Kaplan-Meier survival analysis, PRAME+ status was associated with shorter time to metastasis for both Class 1 and Class 2 tumors together (*P* = 0.0002; 22 metastatic events; median follow-up of 19 months, range 0–125 months) and for Class 2 tumors alone (*P* = 0.01; 15 metastatic events; median follow-up of 18 months, range 0–89 months)(Figure 3A–3B). Similarly, PRAME+ status was associated with shorter time to melanoma-specific mortality for both Class 1 and Class 2 tumors together (*P* = 0.001; 32 melanoma-specific mortality events; median follow-up of 19 months, range 0–142 months) and for Class 2 tumors alone (*P* = 0.02; 28 melanoma-specific mortality events, median follow-up of 18 months, range 0–89 months) (Figure 3C–3D).

### Association between PRAME and chromosomal alterations

**Figure 4:**
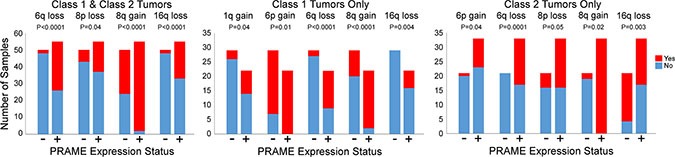
Association of *PRAME* expression with chromosomal gains and losses The bar graphs depict chromosomal gains and losses that were significantly associated with PRAME+ tumors when Class 1 and Class 2 tumors were analyzed together, and when each class was analyzed separately. PRAME+ (red), PRAME− (blue).

To identify chromosomal copy number changes that may be associated with *PRAME* expression, we analyzed 26 of our cases and 80 TCGA cases for which chromosomal copy number and *PRAME* expression data were available (Supplementary Table S3). Overall, PRAME+ tumors were strongly associated with 6q loss (*P* < 0.0001), 8p loss (*P* = 0.04), 8q gain (*P* < 0.0001) and 16q loss (*P* < 0.0001) (Figure 4). Notably, *PRAME* expression status was not associated with monosomy 3 (*P* = 0.3), the chromosomal alteration most strongly associated with metastasis in uveal melanoma, highlighting the potential benefit of including *PRAME* expression status in a prognostic test.

We then analyzed Class 1 and Class 2 tumors separately. Among Class 1 tumors, PRAME+ status was associated with 1q gain (*P* = 0.04), 6p gain (*P* = 0.01), 6q loss (*P* < 0.0001), 8q gain (*P <* 0.0001), and 16q loss (*P* = 0.004). PRAME+ status in Class 2 tumors was associated with 6p gain (*P* = 0.04), 6q loss (*P* = 0.0001), 8p loss (*P* = 0.05), 8q gain (*P* = 0.02) and 16q loss (*P* = 0.003). *PRAME* expression was not associated with monosomy 3 in either comparison (Supplementary Table S3).

### Association between PRAME and driver gene mutations

To identify common driver mutations that may be associated with PRAME+ status, we analyzed 59 of our cases for which mutation data were available, as well as the 80 TCGA cases, for mutations in *EIF1AX*, *BAP1*, *GNA11*, *GNAQ* and *SF3B1* (Supplementary Table S4). When Class 1 and Class 2 tumors were considered together, PRAME+ status was associated with *BAP1* mutations (*P* = 0.02). However, this association is likely due to *BAP1* mutations occurring almost exclusively in Class 2 tumors [9], which we show here to be associated with PRAME+ status. When Class 1 tumors were analyzed separately, *PRAME* expression was directly associated with *SF3B1* mutations (*P* < 0.0001) and inversely associated with *EIF1AX* mutations (*P* = 0.004). There were no mutations associated with *PRAME* expression in Class 2 tumors when analyzed separately.

### *PRAME* expression is associated with aberrant promoter hypomethylation

**Figure 5:**
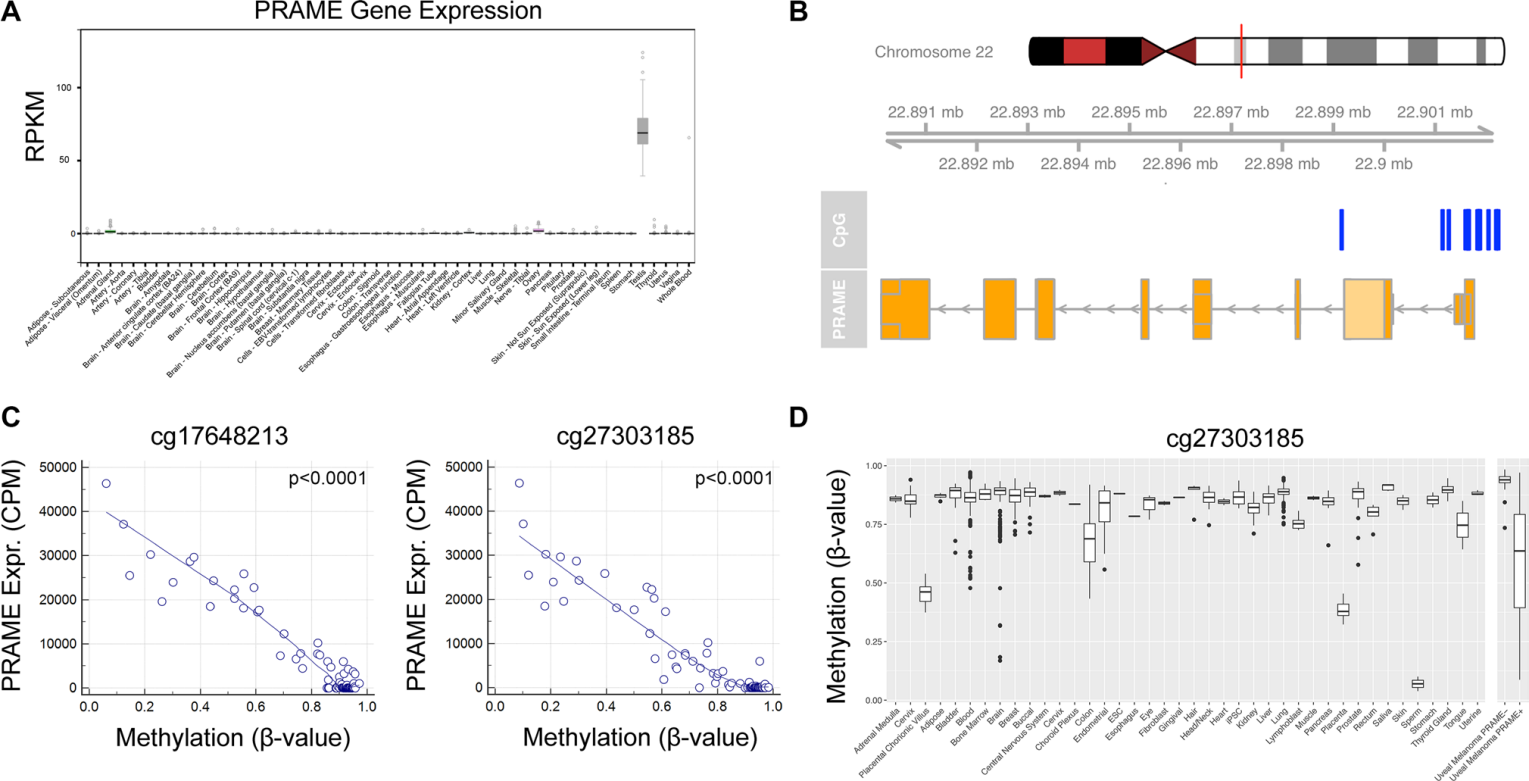
Transcriptional activation of *PRAME* is associated with hypomethylation of the *PRAME* promoter in uveal melanoma (**A**) The only normal adult human tissue that expresses high levels of *PRAME* mRNA is testis. Data were obtained through the GTEx Portal [41]. (**B**) Locations of 12 CpG sites (blue bars) within or near the *PRAME* promoter that exhibited significantly decreased methylation in PRAME+ uveal melanomas (*n* = 41) compared to PRAME- samples (*n* = 39) at a significance level of FDR < 0.05. (**C**) Scatter plots showing the relationship between *PRAME* mRNA expression levels (obtained from TCGA RNA-Seq data) and *PRAME* promoter methylation (obtained from TCGA Infinium HumanMethylation450 BeadChip data) using two representative methylation probes (cg17648213 and cg27303185). Spearman's rank correlation coefficient was used to determine *P*-values. Graphs depicting the other 10 differentially methylated probes are in Supplementary Figure S1. (**D**) Methylation data for the cg27303185 methylation probe was plotted for normal tissues obtained from Marmal-aid [40]. A separate panel (right) depicts PRAME+ and PRAME- uveal melanomas samples for comparison. RPKM, reads per kilobase of transcript per million mapped reads; CPM, counts per million.

Testes is the only normal adult tissue that expresses *PRAME* mRNA at appreciable levels (Figure 5A), which strongly suggests that the expression of *PRAME* in uveal melanoma is anomalous. Consequently, we hypothesized that *PRAME* may become aberrantly activated in uveal melanoma by hypomethylation of the promoter region. Consistent with this hypothesis, 12 CpG sites within and near the *PRAME* promoter were significantly hypomethylated (FDR < 0.05 for all probes) in PRAME+ tumors compared to PRAME− tumors (Figure 5B). We validated these findings using bisulfite conversion followed by Sanger sequencing in a subset of cases (Supplementary Table S5). Strikingly, there was a highly significant correlation between the level of hypomethylation of all 12 CpG sites and the level of mRNA expression (*P* < 0.0001) (Figure 5C and Supplementary Figure S1). The most differentially methylated CpG site (recognized by probe cg27303185) is hypermethylated in all adult tissues except placenta and sperm (Figure 5D). These data indicate that the *PRAME* promoter region is normally hypermethylated and silenced in virtually all normal adult tissues, but it is targeted for hypomethylation and aberrant transcriptional activation during uveal melanoma progression.

## DISCUSSION

We previously reported that *PRAME* mRNA expression is a significant risk factor for metastasis in Class 1 uveal melanomas, and we developed a general method for establishing a PRAME+ threshold in various datasets [6]. In that article, our analysis included a much greater proportion of large tumors treated by enucleation than are encountered in actual clinical practice. However, since we show here that *PRAME* expression is strongly associated with larger tumor size, a study composed primarily of large tumors may not accurately reflect the true range of *PRAME* expression. To pursue the development of *PRAME* as a clinical biomarker, we sought here to rigorously establish a standard method for defining the PRAME+ expression threshold using a standardized and widely used qPCR platform. To achieve a widely applicable threshold and avoid potential systematic biases arising from a single center study, we analyzed a large number of samples obtained from many different ocular oncology centers representing a wide range of tumor sizes and both Class 1 and Class 2 tumors in proportions representative of actual clinical practice. From this analysis, we established a PRAME+ threshold and used it to identify clinical, chromosomal and mutational features associated with *PRAME* expression. We also established a PRAME+ threshold for RNA-Seq using the TCGA dataset, but this threshold must be considered provisional since that dataset was composed primarily of very large tumors treated by enucleation. Indeed, 43% of the TCGA samples were PRAME+, compared to only 27% of our samples.

Across all samples, larger tumor size was the only clinical feature that correlated with PRAME+ status, suggesting that *PRAME* becomes transcriptionally activated later during tumor progression. Interestingly, even though PRAME+ status was a stronger predictor of metastasis in Class 1 tumors, it was also associated with metastasis in Class 2 tumors. In our earlier study that was much smaller and biased towards larger tumors treated by enucleation, we did not find a correlation between *PRAME* expression status and the “1A/1B” system that the clinical test currently uses to indicate low (1A) versus moderate (1B) metastatic risk [6]. In the present study that included a much larger number of samples that better represented the full spectrum of uveal melanomas, we observed a highly significant correlation between PRAME+ status and “1B” status. Nevertheless, since there were a number of discordant cases, we are preparing to start a multi-center prospective study to determine the relative prognostic value of *PRAME* expression status versus the 1A/1B designation in Class 1 tumors in order to determine the optimal biomarker for increased metastatic risk in Class 1 tumors. A limitation of this analysis is the limited follow-up, particularly from the TCGA dataset, which results in a large number of censored data points. Our planned prospective multicenter study with long follow-up is the appropriate study design to validate these findings. Since we found that PRAME+ correlates significantly with tumor size, this multi-center study will also evaluate whether there is a minimum threshold tumor size at which point *PRAME* becomes prognostic.

*PRAME* expression was associated with specific chromosomal gains and losses, some of which were specific to either Class 1 or Class 2 tumors. Changes that were associated with PRAME+ status in both Class 1 and Class 2 tumors included 6p gain, 6q loss, 8q gain and 16q loss. 6p gain and 6q loss were frequently found in the same tumor samples, likely representing the formation of an isochromosome 6p [13, 14]. A previous study identified 16q loss in 16% of uveal melanomas, but no prognostic significance was found [14]. Our study using a larger number of samples and more accurate molecular analytical methods indicates that 16q loss may indeed have prognostic significance. 1q gain was associated with PRAME+ status only in Class 1 tumors, which confirms our previous observation [6]. 1q gain has only rarely been mentioned in the uveal melanoma literature [15], but our findings suggest the need for further studies to determine whether 1q gain has pathogenic as well as prognostic significance. 8p loss was associated with PRAME+ status only in Class 2 tumors, whereas 8q gain was associated with PRAME+ status in both tumor classes. 8q gain is prevalent in both Class 1 and Class 2 tumors, but the mechanism leading to 8q gain tends to be different between the two tumor classes [16]. In Class 1 tumors, 8q gain often occurs through gain of an entire copy of chromosome 8 or by simple gain of the q arm, whereas in Class 2 tumors, 8q gain frequently occurs through formation of an isochromosome 8q, which is accompanied by loss of 8p [17]. The common association of *PRAME* expression and isochromosome formation on chromosomes 6 and 8 is of interest and may provide new insight into uveal melanoma tumorigenesis. We previously showed that genes which become aberrantly up-regulated in PRAME+ tumors are enriched for functions related to chromosome maintenance, meiotic recombination and telomere maintenance [6]. In addition, the PRAME protein has been shown to associate at transcriptional target sites on chromatin with the KEOPS/EKC complex [18], which is involved in chromosome segregation, telomere maintenance and other highly conserved functions [19]. Hence, aberrant expression of *PRAME* may predispose tumor cells to isochromosome formation, as well as other forms of aneuploidy that promote tumor progression.

Our finding that *PRAME* becomes aberrantly hypomethylated and transcriptionally activated during uveal melanoma progression is similar to findings in other cancers [20, 21] and may have therapeutic implications. Since *PRAME* is not normally expressed in most normal adult tissues, targeted molecular inhibition of the PRAME protein or immunotherapy directed against PRAME−expressing tumor cells may be well tolerated. Indeed, there is growing evidence that PRAME may be a good target for immunotherapy [22–25]. Since the PRAME protein is not normally expressed on the cell surface, one strategy is to target PRAME using a T-cell receptor mimic (TCRm) monoclonal antibody that recognizes the PRAME^300–309^ peptide presented by HLA*A02:01 on the cell surface [26]. Others have developed PRAME−specific cytotoxic T lymphocytes that have shown effective responses against PRAME-expressing tumor cells, including progenitor populations that are notoriously resistant to current cancer therapeutic strategies [24, 27]. Furthest along in development are vaccines against PRAME that are currently undergoing clinical trials in cutaneous melanoma and other cancers (Trial numbers NCT01149343, NCT01853878 and NCT00423254) [28]. Interestingly, we evaluated *PRAME* expression of two matched primary and metastatic UM samples analyzed by the Illumina HumanRef-8 v1.0 expression microarray in our previously published dataset (GEO accession number GSE39717) [29], and we found that both the primary and metastatic samples were PRAME+ (data not shown), supporting a mechanistic role for PRAME expression in UM metastasis. Since no effective therapies currently exist for metastatic uveal melanoma [30], our center and others are preparing to undertake clinical trials to assess the efficacy of PRAME-directed immunotherapy in appropriately selected patients.

In summary, we have provided a threshold for PRAME+ expression from qPCR data for primary uveal melanomas across a wide spectrum of tumor sizes and in both tumor classes representative of actual clinical practice. We previously identified *PRAME* expression as a biomarker for increased metastatic risk in Class 1 tumors [6], and here we showed for the first time that *PRAME* expression is also associated with worse prognosis among Class 2 tumors. We demonstrated that specific chromosomal gains and losses, as well as specific driver mutations, are found preferentially in PRAME+ tumors. Finally, we showed that specific CpG sites around the *PRAME* promoter are differentially hypomethylated in PRAME+ tumors, suggesting that the aberrant transcriptional activation of *PRAME* in uveal melanoma is the result of epigenetic reprogramming during tumor progression. In addition to its prognostic value, *PRAME* expression status may potentially be useful in the future for guiding the use of PRAME-directed immunotherapy, which would make PRAME the first true “companion prognostic” biomarker in uveal melanoma.

## MATERIALS AND METHODS

The sources of all uveal melanoma samples used in this study are summarized in Supplementary Table S6. Tumor samples were obtained from 123 primary uveal melanomas from the practice of one of the authors (JWH), including 64 samples that were included in a previous publication [6]. The research was conducted in a HIPAA-compliant manner in accordance with the tenets of the Declaration of Helsinki. Approval was obtained from the Institutional Review Board of the University of Miami. Written informed consent was obtained from each patient from our center. Baseline clinical information and patient outcomes were recorded. De-identified *PRAME* expression and GEP Class data were obtained from 555 uveal melanoma samples from Castle Biosciences that had been collected between July 21, 2015, and March 2, 2016, as part of internal *PRAME* qPCR method development. These samples were obtained as formalin-fixed paraffin-embedded tissue from enucleations in 55 (9.9%) cases and as fresh-frozen samples from fine needle aspirate biopsies in 500 (90.1%) cases. The data available for these cases included GEP class, 1A versus 1B subtype for Class 1 tumors, and *PRAME* mRNA expression. Additionally we analyzed clinical, whole exome sequencing, RNA sequencing, SNP 6.0 array data, and DNA methylation data from 80 uveal melanoma samples generated by the TCGA Research Network: http://cancergenome.nih.gov/.

### PRAME mRNA expression analysis

For the RNA samples from our center and from Castle Biosciences, *PRAME* mRNA expression was analyzed by qPCR using the Applied Biosystems 7900 HT Real-Time PCR System with TaqMan primers and Gene Expression Master Mix following the manufacturer's protocol as previously described [6]. Ct values were calculated using the manufacturer's software and ΔCt values were calculated by subtracting the geometric mean of the Ct values of the endogenous control genes from the mean Ct values for *PRAME*, as previously described [6]. Relative normal expression was calculated using the equation 2^-ΔCT. For the 80 samples from the TCGA, raw RNA-Seq datasets were aligned to the hg19 genome using STAR [31], which was also used to generate count files. Count files were then normalized using DeSeq2 [32]. Next-generation sequencing analysis was conducted on Pegasus, the supercomputer administered by the Advanced Computing Group of the Center for Computational Science at the University of Miami. For *PRAME* mRNA expression in normal tissues, RNA-Seq data was obtained from the Genotype-Tissue Expression (GTEx) project [33].

### Estimating class status from RNA-sequencing data

For research purposes of this study, we estimated the gene expression profile class assignment for the 80 TCGA samples, which were analyzed by RNA-Seq. Raw RNA-Seq datasets were prepared using the pipeline described in the previous section. The top 20% most variable genes were selected, analyzed by principal component analysis, and plotted using the stats, matrixstats, and rgl packages, respectively, in R (version 3.2.3). This analysis grouped the samples into two clusters, as we have previously described for Class 1 and Class 2 tumors [34]. The identity of each cluster was determined to be most consistent with Class 1 versus Class 2 based on the expression of genes previously known to be differentially up-regulated in each Class. The DecisionDx-UM test results were available for 11 of these samples, and there was 100% concordance with our class assignment. This method was used solely for research purposes and is not meant for actual clinical testing, as it has not been prospectively validated in a manner analogous to the DecisionDx-UM test.

### Determining PRAME+ expression threshold

qPCR and RNA-Seq samples were separately ordered from lowest to highest relative and normalized *PRAME* expression, respectively, and each was plotted with a line representing the best-fitting LOESS model (second degree, family = ”Gaussian”, spanning 0.4 for qPCR and 0.45 for RNA-Seq, fitting by least-squares) (Figure 1A). Based on the LOESS model, a predicted dataset fitting the LOESS model was generated (Figure 1B) and the slope between each predicted point was calculated and plotted (Figure 1C) to represent the change in slope. The point of inflection where the slope sustainably rose above baseline was defined as the cut-off for PRAME+ and PRAME− (Figure 1C–1D).

### Exome sequencing and chromosomal copy number analysis

Whole-exome sequencing was conducted on 24 of our primary uveal melanomas and matched blood using NimbleGen SeqCap EZ Human Exome Library v2.0 (Roche Nimblegen) and run on the Illumina Genome Analyzer II. Exome sequencing data on 80 primary uveal melanoma TCGA samples were downloaded from CGHub and aligned to the hg19 reference genome using Novoalign. Variant calling was conducted using Mutect2 [35] and Varscan2 [36]. Chromosomal copy number analysis was obtained for 106 samples, including 26 samples from our center (15 from previously published data and 11 newly analyzed from exome sequencing data) and 80 from the TCGA. Chromosomal gains and losses were called by CNVKit [37] for exome sequencing data and by ASCAT [38] for TCGA SNP 6.0 array data.

### DNA methylation analysis

The 80 TCGA uveal melanoma tumors samples were assayed for global DNA methylation status with the Infinium HumanMethylation450K BeadChip (Illumina). This kit interrogates ∼450,000 methylation sites at single-nucleotide resolution, including at CpG sites within promoter, 5′UTR, first exon, gene body, and 3′UTR regions. Methylation data underwent quality control, normalization, and differential analysis of PRAME+ and PRAME− samples using the ChAMP methylation pipeline in R [39]. CpG sites that were differentially hypomethylated at a significance level of FDR < 0.05 were plotted along the *PRAME* locus using the GViz package in R. All 12 methylation probes targeting *PRAME* that are included in the Methyl450K array were significantly hypomethylated in the TCGA PRAME+ samples.

For validation, primers were designed against a region containing 3 of these 12 probes and validated in 4 PRAME+ and 3 PRAME− samples. This validation study was small due to limited sample availability. For primer design, 500 ng of tumor DNA was bisulfite converted using the EZ Methylation-Lightning Kit (Zymo Research). Primers for PCR amplification of the *PRAME* promoter were designed with the Bisulfite Primer Seeker (http://www.zymoresearch.com/tools/bisulfite-primer-seeker). Forward Primer: GAAGGATTTCGTGTTTAAGGTTTTTTAAGG. Reverse Primer: GTGTTTTTATTTTGGAAATAGAGATTTAGT TTTTTTT. The *PRAME* promoter region was amplified with the EpiMark Hot Start Taq polymerase (New England Biolabs) at Tm = 54.5°C, and the PCR product purified by agarose gel separation/elution before Sanger sequencing. The status of the *PRAME* methylation site detected by Infinium HumanMethylation450K BeadChip probe cg27303185 in normal tissues was obtained from Marmal-aid [40] and plotted in a box-and-whisker with ggplot2 in R in comparison to TCGA uveal melanoma data.

### Statistical analysis

Statistical analysis was performed using Medcalc^®^ version 14.10.2. Fisher's exact test was used to evaluate discrete dichotomous variables, the Mann-Whitney test for comparison of continuous variables, Spearman's rho for correlation analyses of continuous variables, and Kaplan-Meier survival analysis for determining the association of *PRAME* expression status with patient outcomes.

## SUPPLEMENTARY MATERIALS TABLES AND FIGURE


