# Supplementary material for: Epigenetic reprogramming and aberrant expression of PRAME are associated with increased metastatic risk in Class 1 and Class 2 uveal melanomas
Source: Oncotarget. 2016 Jul 30;7(37):59209–19. doi: 10.18632/oncotarget.10962 (PMC5312306; doi:10.18632/oncotarget.10962)
Supplement: Supplementary file 1 [file oncotarget-07-59209-s001.pdf]

Epigenetic reprogramming and aberrant expression of PRAME are associated with increased metastatic risk in Class 1 and Class 2 uveal melanomas

Supplementary Materials

Supplementary Table S1: Association of clinical features with *PRAME* status in 123 uveal melanoma cases from our practice

Variable	All Classes (*n* = 123)	Association with PRAME+ (*P*-value)	Class 1 (*n* = 63)	Association with PRAME+ (*P*-value)	Class 2 (*n* = 60)	Association with PRAME+ (*P*-value)
Age at diagnosis, y<br>Mean<br>Median (Q1–Q3)	 62.4<br>64.6 (52.2–74.0)	0.3	 58.0<br>61.0 (48.2–69.2)	0.1	 67.1<br>68.7 (61.1–76.0)	0.7
Sex, No.(%)<br>Female<br>Male	 67.0 (54.5)<br>56.0 (45.5)	0.6	 39.0 (61.9)<br>24.0 (38.1)	0.8	 28.0 (46.7)<br>32.0 (53.3)	0.8
Ciliary body involvement, No. (%)<br>Yes<br>No<br>Not Available	 58.0 (47.2)<br>63.0 (51.2)<br>2.0 (1.6)	0.2	 22.0 (35.5)<br>40.0 (64.5)<br>0.0 (0.0)	0.8	 36.0 (60.0)<br>23.0 (38.3)<br>1.0 (1.7)	0.06
Extra-ocular extension, No. (%)<br>Yes<br>No<br>Not Available	 17.0 (13.8)<br>92.0 (74.8)<br>14.0 (11.4)	> 0.9	 4.0 (6.4)<br>54.0 (85.7)<br>5.0 (7.9)	0.6	 13.0 (21.7)<br>38.0 (63.3)<br>9.0 (15.0)	> 0.9
LBD, mm<br>Mean<br>Median (Q1-Q3)	 15.5<br>16.0 (12.0–19.1)	0.01	 14.7<br>14.0 (11.0–18.0)	0.03	 16.3<br>17.0 (14.0–19.8)	0.1
Tumor thickness, mm<br>Mean<br>Median (Q1–Q3)	 9.5<br>10.0 (6.4–12.0)	0.02	 8.8<br>10.0 (6.9–12.0)	0.7	 10.1<br>11.0 (8.1–12.7)	0.002
Metastasis, No. (%)<br>Yes<br>No<br>Not Available	 28.0 (22.8)<br>95.0 (77.2)<br>0.0 (0.0)	NA	 8.0 (12.7)<br>55.0 (87.3)<br>0.0 (0.0)	NA	 20.0 (33.3)<br>40.0 (66.7)<br>0.0 (0.0)	NA
Melanoma-specific mortality, No. (%)<br>Yes<br>No<br>Not Available	 21.0 (17.1)<br>102.0 (82.9)<br>0.0 (0.0)	NA	 4.0 (6.3)<br>59.0 (93.7)<br>0.0 (0.0)	NA	 17.0 (28.3)<br>43.0 (71.7)<br>0.0 (0.0)	NA
All-cause mortality, No (%)<br>Yes<br>No<br>Not Available	 23.0 (18.7)<br>100.0 (81.3)<br>0.0 (0.0)	NA	 5.0 (7.9)<br>58.0 (92.1)<br>0.0 (0.0)	NA	 18.0 (30.0)<br>42.0 (70.0)<br>0.0 (0.0)	NA
Time to last follow-up or death, mo<br>Mean<br>Median (Q1–Q3)	 29.6<br>21.0 (13.0–33.5)	NA	 37.3<br>22.0 (13.5–54.0)	NA	 9.2<br>20.0 (10.8–26.1)	NA

NA, not applicable (Kaplan-Meier survival analysis shown in article).

Supplementary Table S2: Association of clinical features with *PRAME* status in 80 TCGA uveal melanoma cases

Variable	All Classes (*n* = 80)	Association with PRAME+ (*P*-value)	Class 1 (*n* = 39)	Association with PRAME+ (*P*-value)	Class 2 (*n* = 41)	Association with PRAME+ (*P*-value)
Age at diagnosis, y<br>Mean<br>Median (Q1–Q3)	 61.7<br>61.5 (51.0–74.3)	0.2	 58.5<br>60.0 (50.5–71.0)	0.04	 64.7<br>64.0 (55.0–76.0)	> 0.9
Sex, No.(%)<br>Female<br>Male	 35.0 (43.8)<br>45.0 (56.2)	> 0.9	 17.0 (43.6)<br>22.0 (56.4)	> 0.8	 18.0 (43.9)<br>23.0 (56.1)	> 0.8
Ciliary body involvement, No. (%)<br>Yes<br>No<br>Not Available	 24.0 (30.0)<br>56.0 (70.0)<br>0.0 (0.0)	> 0.9	 8.0 (13.1)<br>31.0 (50.8)<br>22.0 (36.1)	> 0.7	 16.0 (39.0)<br>25.0 (61.0)<br>0.0 (0.0)	> 0.5
Extra-ocular extension, No. (%)<br>Yes<br>No<br>Not Available	 7.0 (8.8)<br>68.0 (85.0)<br>5.0 (6.2)	0.4	 1.0 (2.6)<br>36.0 (92.3)<br>2.0 (5.1)	> 0.9	 6.0 (14.6)<br>32.0 (78.1)<br>3.0 (7.3)	> 0.2
LBD, mm<br>Mean<br>Median (Q1–Q3)	 16.9<br>17.0 (14.8–19.4)	0.02	 16.3<br>16.5 (14.0–18.5)	0.4	 17.6<br>17.5 (15.5–20.0)	> 0.03
Tumor thickness, mm<br>Mean<br>Median (Q1–Q3)	 10.4<br>10.5 (8.5–12.0)	0.2	 10.0<br>10.0 (6.9–12.0)	> 0.9	 10.8<br>11.0 (8.1–12.7)	> 0.1
Metastasis, No. (%)<br>Yes<br>No<br>Not Available	 12.0 (15.0)<br>65.0 (81.2)<br>3.0 (3.8)	NA	 0.0 (0.0)<br>38.0 (97.4)<br>1.0 (2.6)	NA	 12.0 (29.3)<br>27.0 (65.8)<br>2.0 (4.9)	NA
Melanoma-specific mortality, No. (%)<br>Yes<br>No<br>Not Available	 11.0 (13.8)<br>68.0 (85.0)<br>1.0 (1.2)	NA	 0.0 (0.0)<br>39.0 (62.9)<br>23.0 (37.1)	NA	 11.0 (26.8)<br>29.0 (70.7)<br>1.0 (2.5)	NA
All-cause mortality, No (%)<br>Yes<br>No<br>Not Available	 13.0 (16.2)<br>67.0 (83.8)<br>0.0 (0.0)	NA	 1.0 (2.6)<br>38.0 (97.4)<br>0.0 (0.0)	NA	 12.0 (29.3)<br>29.0 (70.7)<br>0.0 (0.0)	NA
Time to last follow-up or death, mo<br>Mean<br>Median (Q1–Q3)	 15.0<br>12.9 (2.4–23.4)	NA	 17.4<br>13.3 (2.4–25.3)	NA	 7.8<br>12.8 (2.7–19.7)	NA

NA, not applicable (Kaplan-Meier survival analysis shown in article).

Supplementary Table S3: Associations between *PRAME* status and copy number gains and losses in 106 Class 1 and Class 2 uveal melanomas

Variable	All Classes (*n* = 106)	Association with PRAME+ (*P*-value)	Class 1 (*n* = 51)	Association with PRAME+ (*P*-value)	Class 2 (*n* = 55)	Association with PRAME+ (*P*-value)
1p loss, No. (%)						
Yes	33 (31.1)	0.7	11 (21.6)	> 0.9	22 (40.0)	> 0.9
No	73 (68.9)		40 (78.4)		33 (60.0)	
Not Available	0 (0.0)		0 (0.0)		0 (0.0)	
1q gain, No. (%)						
Yes	18 (17.0)	0.07	11 (21.6)	0.04	7 (12.7)	> 0.9
No	88 (83.0)		40 (78.4)		48 (87.3)	
Not Available	0 (0.0)		0 (0.0)		0 (0.0)	
Chr3 loss, No. (%)						
Yes	53 (50.0)	0.3	0 (0.0)	0.5	53 (96.4)	0.5
No	53 (50.0)		51 (100.0)		2 (3.6)	
Not Available	0 (0.0)		0 (0.0)		0 (0.0)	
6p gain, No. (%)						
Yes	55 (51.9)	0.1	44 (86.3)	0.02	11 (20.0)	0.05
No	51 (48.1)		7 (13.7)		44 (80.0)	
Not Available	0 (0.0)		0 (0.0)		0 (0.0)	
6q loss, No. (%)						
Yes	32 (30.2)	< 0.0001	15 (29.4)	< 0.0001	17 (30.9)	< 0.0001
No	74 (69.8)		33 (70.6)		38 (69.1)	
Not Available	0 (0.0)		0 (0.0)		0 (0.0)	
8p loss, No. (%)						
Yes	25 (23.6)	0.02	3 (5.9)	> 0.9	22 (40.0)	0.03
No	81 (76.4)		48 (94.1)		33 (60.0)	
Not Available	0 (0.0)		0 (0.0)		0 (0.0)	
8q gain, No. (%)						
Yes	80 (75.5)	< 0.0001	29 (56.9)	< 0.0001	51 (92.7)	0.07
No	26 (24.5)		22 (43.1)		4 (7.3)	
Not Available	0 (0.0)		0 (0.0)		0 (0.0)	
16q loss, No. (%)						
Yes	24 (22.6)	< 0.0001	6 (11.8)	0.004	18 (32.7)	0.01
No	82 (77.4)		45 (88.2)		37 (67.3)	
Not Available	0 (0.0)		0 (0.0)		0 (0.0)	

Supplementary Table S4: Associations between *PRAME* status and common driver mutations in 139 Class 1 and Class 2 uveal melanomas

Variable	All Classes (*n* = 139)	Association with PRAME+ (*P*-value)	Class 1 (*n* = 63)	Association with PRAME+ (*P*-value)	Class 2 (*n* = 60)	Association with PRAME+ (*P*-value)
BAP1, No. (%)						
Yes	60 (43.2)	0.02	1 (1.6)	0.5	59 (77.6)	0.2
No	64 (46.0)		54 (85.7)		10 (13.2)	
Not Available	15 (10.8)		8 (12.7)		7 (9.2)	
SF3B1, No. (%)						
Yes	28 (20.1)	0.05	21 (33.3)	< 0.0001	7 (9.2)	0.4
No	88 (63.3)		35 (55.6)		53 (69.7)	
Not Available	23 (16.6)		7 (11.1)		16 (21.1)	
EIF1AX, No. (%)						
Yes	16 (11.5)	0.02	14 (22.2)	0.004	2 (2.6)	0.5
No	90 (64.8)		38 (60.3)		52 (68.4)	
Not Available	33 (23.7)		11 (17.5)		22 (29.0)	
GNAQ, No. (%)						
Yes	67 (48.2)	0.5	38 (60.3)	0.6	29 (28.7)	0.3
No	64 (46.0)		24 (38.1)		40 (52.6)	
Not Available	8 (5.8)		1 (1.6)		7 (9.2)	
GNA11, No. (%)						
Yes	55 (39.6)	> 0.9	20 (31.7)	> 0.9	35 (46.1)	> 0.9
No	74 (53.2)		41 (65.1)		33 (43.4)	
Not Available	10 (7.2)		2 (3.2)		8 (10.5)	
CYSLTR2, No. (%)						
Yes	3 (2.2)	NA	1 (1.6)	NA	2 (2.6)	NA
No	77 (55.4)		38 (60.3)		39 (51.3)	
Not Available	59 (42.4)		24 (38.1)		35 (46.1)	
PLCB4, No. (%)						
Yes	2 (1.4)	NA	0 (0.0)	NA	2 (3.3)	NA
No	78 (56.1)		39 (61.9)		23 (38.3)	
Not Available	59 (42.4)		24 (38.1)		35 (58.4)	

NA, not applicable (insufficient number of cases for statistical analysis).

Supplementary Table S5: Validation of differential methylation at 5 CpG sites within the *PRAME* promoter region in PRAME+ versus PRAME- tumors

Genomic location on chromosome 22

		22901898	22901907	22902166	22902216	22902255
PRAME+ tumors	MM10	X	X		X	X
	MM92			X	X	
	MM89					X
	MM16					
PRAME− tumors	MM28					
	MM49					
	MM50					

X, indicates hypomethylation of indicated nucleotide.

Supplementary Table S6: Sources and numbers of uveal melanoma samples for the various data analyses

Variable	Our Practice	Castle	TCGA
Number of samples	123	555	80
Class 1/Class 2 data	Yes	Yes	Estimated
Class 1A/1B data	Yes	Yes	No
Clinical annotations	Yes	No	Yes
Whole-exome data	24	No	80
Mutation data	59	No	80
Chromosomal copy number data	26	No	80

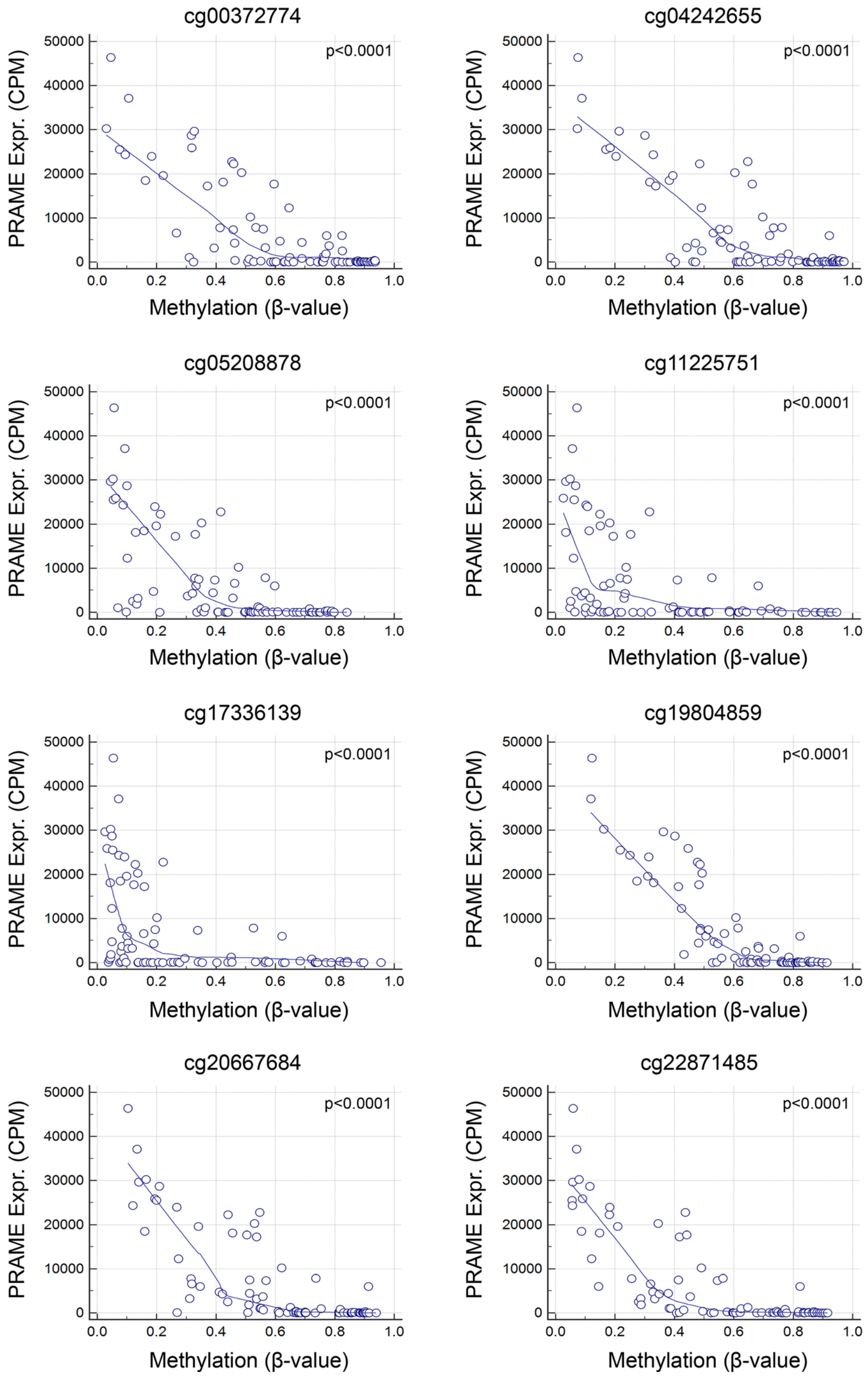

Supplementary Figure S1: Association between PRAME mRNA expression and promoter hypomethylation. Scatter plots showing the relationship between PRAME mRNA expression levels (obtained from the TCGA RNA-Seq data) and PRAME promoter methylation (obtained from the TCGA Infinium Human Methylation 450 Bead Chip Array data) at ten additional methylation sites represented by the indicated probes. Spearman's rank correlation coefficient was used to determine *P*-values.
